# The status of hepatitis B control in the African region

**DOI:** 10.11604/pamj.supp.2017.27.3.11981

**Published:** 2017-06-22

**Authors:** Lucy Breakwell, Carol Tevi-Benissan, Lana Childs, Richard Mihigo, Rania Tohme

**Affiliations:** 1 Global Immunization Division, Centers for Disease Control and Prevention, Atlanta, GA, USA; 2 World Health Organization Regional Office for Africa, Brazzaville, Republic of Congo; 3 Oak Ridge Institute for Science and Education, Centers for Disease Control and Prevention, Atlanta, GA, USA

**Keywords:** Hepatitis B control, vaccination, hepatitis B prevalence

## Abstract

The World Health Organization (WHO) African Region has approximately 100 million people with chronic hepatitis B virus (HBV) infection. This review describes the status of hepatitis B control in the Region. We present hepatitis B vaccine (HepB) coverage data and from available data in the published literature, the impact of HepB vaccination on hepatitis B surface antigen (HBsAg) prevalence, a marker of chronic infection, among children, HBsAg prevalence in pregnant women, and risk of perinatal transmission. Lastly, we describe challenges with HepB birth dose (HepB-BD) introduction reported in the Region, and propose strategies to increase coverage. In 2015, regional three dose HepB coverage was 76%, and 16(34%) of 47 countries reported ≥ 90% coverage. Overall, 11 countries introduced HepB-BD; only nine provide universal HepB-BD, and of these, five reported ≥ 80% coverage. From non-nationally representative serosurveys among children, HBsAg prevalence was lower among children born after HepB introduction compared to those born before HepB introduction. However, some studies still found HBsAg prevalence to be above 2%. From limited surveys among pregnant women, the median HBsAg prevalence varied by country, ranging from 1.9% (Madagascar) to 16.1% (Niger); hepatitis B e antigen (HBeAg) prevalence among HBsAg-positive women ranged from 3.3% (Zimbabwe) to 28.5% (Nigeria). Studies in three countries indicated that the risk of perinatal HBV transmission was associated with HBeAg expression or high HBV DNA viral load. Major challenges for timely HepB-BD administration were poor knowledge of or lack of national HepB-BD vaccination guidelines, high prevalence of home births, and unreliable vaccine supply. Overall, substantial progress has been made in the region. However, countries need to improve HepB3 coverage and some countries might need to consider introducing the HepB-BD to help achieve the regional hepatitis B control goal of < 2% HBsAg prevalence among children < 5 years old by 2020. To facilitate HepB-BD introduction and improve timely coverage, strategies are needed to reach both facility-based and home births. Strong political commitment, clear policy recommendations and staff training on HepB-BD administration are also required. Furthermore, high quality nationally representative serosurveys among children are needed to inform decision makers about progress towards the regional control goal.

## Introduction

About 100 million persons in the World Health Organization (WHO) AfricanRegion have chronic hepatitis B virus (HBV) infection, and all countries in the Region have an intermediate (2%–7%) or high (≥ 8%) population prevalence of chronic HBV infection [1, 2]. Chronically infected individuals have a 15%–25% estimated lifetime risk of developing liver cancer or cirrhosis, dependent upon age at infection [1]. About 70%–90% of infants infected before 1 year of age will develop chronic HBV infection, compared with 20%–50% of those infected between 1-5 years of age, and with 5%–10% of those infected after 5 years of age [1]. About 90% of babies born to hepatitis B surface antigen (HBsAg) positive (a serologic marker of chronic HBV infection) and hepatitis B e antigen (HBeAg) positive (a marker of infectivity) mothers become chronically infected, compared with about 35% of babies born to HBeAg-negative chronically infected mothers [1]. In areas of intermediate or high endemicity, the majority of chronic HBV infections in the population are attributable to mother-to-child (perinatal) and early childhood transmission [1]. Childhood transmission is effectively prevented by administration of hepatitis B vaccine (HepB) in the routine childhood vaccination schedule, and perinatal transmission is effectively prevented by the timely administration of a HepB birth dose (HepB-BD). WHO recommends that all infants receive hepatitis B vaccine at birth, preferably within 24 hours, followed by two or three additional doses with a minimum interval of four weeks [1].

In November 2014, the WHO African Regional Committee endorsed a resolution for a hepatitis B control goal to reduce chronic HBV infection prevalence to < 2% in children less than 5 years of age in all Members States by 2020 [3]. In this paper, we present the status of hepatitis B control in the Region, including national policies for routine childhood hepatitis B vaccination and HepB-BD, coverage estimates for the HepB series and the HepB-BD, available HBsAg prevalence data among children pre- and post-HepB introduction, and available data on the risk of mother-to-child-transmission of HBV. We also describe common challenges associated with HepB-BD introduction, and propose strategies to facilitate increased HepB-BD coverage in the African Region.

## Methods

For each country in the WHO African Region, we compiled data on hepatitis B vaccination (WHO-UNICEF coverage estimates) [4, 5], the number of annual births [6], the proportion of institutional births, the proportion of births attended by a skilled birthing assistant (SBA), and an estimate of at least one antenatal care (ANC) visit [7].

We also conducted a review of published literature from January 1995 to October 2016 using MEDLINE with the search criteria “(Country Name) AND hepatitis B”. The search time frame accounted for women of child bearing age and the exponential rise of HIV cases in the 1980s and early 1990s, which could have affected the risk of HBV transmission if individuals were HBV/HIV co-infected, before introduction of HIV antiviral treatment [8, 9]. Articles were initially selected by title, then by abstract review prior to complete article review. Any article that reported HBsAg prevalence among pregnant women and children, or transmission of HBV from mother to child was considered for inclusion. Articles which included prisoners, commercial sex workers, members of the armed forces, or blood donors were excluded as these groups might have higher or lower HBsAg prevalence compared to the general population, resulting in biased results. We also excluded studies that only tested infant cord blood (as this might have been contaminated with the mother’s blood) as well as studies that only included known HIV-positive women. The latter for two reasons; HIV and HBV share transmission pathways and many HIV positive women in the studies were provided anti-retroviral treatment (which also lowers HBV DNA levels and subsequently decreases the risk of perinatal transmission of HBV). Articles with fewer than 100 participants or that did not specify the laboratory test used to determine HBsAg status were also excluded. We identified additional articles cited in manuscripts identified in the literature search. From over 2,000 articles identified, 99 articles were selected for inclusion following full paper review. For The Gambia, where HepB had been introduced during the early 1990s, and in Cameroon, and Tanzania, where HBsAg prevalence among children pre-HepB introduction were unavailable after 1995, we identified five relevant articles for inclusion that were published from 1990 to1995. Median HBsAg and HBeAg prevalence estimates among pregnant women were calculated for each country using data from relevant studies. Minimum and maximum estimates are also presented.

We also summarized findings from HepB-BD post-introduction assessments conducted in Botswana (Feb 2016), Namibia (May 2016), Nigeria (Sept 2015), and Sao Tome and Principe (Jul 2015) (unpublished reports). For Mauritania, a joint Centers for Disease Control and Prevention (CDC)-WHO trip report (Dec 2015) was used to provide information on the current status of the HepB-BD implementation. Further information about country experiences with HepB-BD was gathered from the literature search described and from the reports of a Hepatitis B Birth Dose Training Workshop (Feb 2015) and the Regional Consultation on Viral Hepatitis Control in the WHO African Region (Nov 2016).

## Current status of knowledge

### Current status of Hepatitis B control in the African region

### Childhood hepatitis B vaccination

All 47 countries in the WHO Africa Region have introduced HepB into the routine infant immunization schedule; 44 (94%) countries use pentavalent vaccine (diphtheria, tetanus, pertussis, Haemophilus influenza type B and hepatitis B vaccines) and 33 (70%) countries follow a three-dose schedule at 6, 10, and 14 weeks of age (Table 1). As of December 2016, nine countries, representing 28% of the regional birth cohort, have introduced auniversal HepB-BD policy (Table 2). Two countries, Sao Tome and Principe and Mauritius, only provide HepB-BD for babies born to HBsAg-positive mothers [10].

**Table 1:** childhood hepatitis B vaccine 3-dose (HepB3) coverage by country in the World Health Organization African Region, 2011–2015

Country^1^	Year Introduced^2^	HepB3 Coverage %^2^				
		2011	2012	2013	2014	2015
Algeria	2001	95	95	95	95	95
Angola	2006	72	75	77	64	64
Benin	2005	75	81	74	75	79
Botswana	1995	95	95	95	95	95
Burkina Faso	2006	91	90	88	91	91
Burundi	2004	96	96	96	95	94
Cabo Verde	2002	90	94	93	95	93
Cameroon	2005	82	85	89	87	84
Central African Republic	2008	47	47	23	47	47
Chad	2008	33	45	48	46	55
Comoros	2003	83	86	83	80	80
Congo	2007	80	79	85	90	80
Cote d’Ivoire	2001	62	82	80	76	83
DR of Congo	2007	74	75	74	80	81
Equatorial Guinea	2013	-	-	-	20	16
Eritrea	2002	96	94	94	94	95
Ethiopia	2007	65	69	72	77	86
Gabon	2004	75	82	79	70	80
Gambia	1990	96	98	97	96	97
Ghana	2002	91	92	90	98	88
Guinea	2006	63	62	63	51	51
Guinea-Bissau	2008	80	80	80	80	80
Kenya	2002	96	94	93	92	89
Lesotho	2003	96	95	93	93	93
Liberia	2008	77	80	76	50	52
Madagascar	2002	73	70	74	73	69
Malawi	2002	97	96	89	91	88
Mali	2003	66	68	71	77	68
Mauritania	2005	75	80	80	84	73
Mauritius	1997	98	98	98	97	97
Mozambique	2001	76	76	78	79	80
Namibia	2009	82	84	89	88	92
Niger	2008	75	71	67	68	65
Nigeria	2004	46	42	45	49	56
Rwanda	2002	97	98	98	98	98
Sao Tome and Principe	2003	96	96	97	95	96
Senegal	2004	92	91	92	89	89
Seychelles	1995	99	99	99	99	98
Sierra Leone	2007	89	91	92	83	86
South Africa	1995	76	73	65	74	71
South Sudan	2014	-	-	-	-	31
Swaziland	1996	91	95	98	98	98
Togo	2008	85	84	84	87	88
Uganda	2002	82	78	78	78	78
United Republic of Tanzania	2002	90	92	91	97	98
Zambia	2005	81	78	79	86	90
Zimbabwe	2000	94	97	95	91	87

1 All countries provide pentavalent (DTwPHibHepB) vaccine, except for Algeria and Mauritius which provide monovalent hepatitis B vaccine and South Africa which provides monovalent and hexavalent (DTaPIPVHibHepB) vaccines. All countries follow a 3-dose schedule at 6, 10, and 14 weeks of age, except for Algeria (0, 1, 5 months), Angola (0, 2, 4, 5 months), Botswana (0, 2, 3, 4 months), Burkina Faso (8, 12, 16 weeks), Cabo Verde (0, 2, 4, 6, 18 months), Congo (8, 12, 16 weeks), Gambia (0, 2, 3, 4 months), Mauritania (0, 6, 10, 14 weeks), Namibia (0, 6, 10, 14 weeks), Nigeria (0, 6, 10, 14 weeks), Sao Tome and Principe (0, 6, 10, 14 weeks), Senegal (0, 6, 10, 14 weeks), Seychelles (3, 4, 5 months), and South Africa (6, 10, 14 weeks, 18 months for hexavalent vaccine).

2 Vaccine introduction year and annual coverage estimates were derived from WHO UNICEF Joint Reporting (updated July 2016) http://apps.who.int/immunization_monitoring/globalsummary/timeseries/tswucoveragehepb3.html.

**Table 2:** Hepatitis B vaccine birth dose (HepB-BD) coverage, institutional births, and antenatal care visits by country in the World Health Organization African Region

Country	Year HepB-BD introduced	HepB-BD Coverage %^1^					Annual Births (1000s)^2^	Institutional deliveries %^3^	Births attended by SBA %^3^	>1 ANC visit %^3^
		2011	2012	2013	2014	2015				
Algeria	2004	99	99	99	99	99	936	97	97	93
Angola^4^	2015	-	-	-	-	19	1,128	46	47	80
Botswana	Pre 2000	99	99	99	99	99	55	100	95	94
Cabo Verde	2002	99	99	94	99	93	11	76	78	98
Gambia	1990	90	97	93	96	98	83	63	57	86
Mauritania	2013	-	-	-	-	51	134	65	65	84
Mauritius^5^	n.a.	-	-	-	-	-	14	99	100	-
Namibia	2014	-	-	-	1	87	72	87	88	97
Nigeria	2004	31	32	32	32	32	7,133	36	38	61
Sao Tome and Principe^5^	2002	-	-	-	-	-	6	91	93	98
Senegal	2016	-	-	-	-	-	567	77	59	96
No birth dose										
Benin							388	87	77	83
Burkina Faso							717	66	66	94
Burundi							488	60	60	99
Cameroon							847	61	65	83
Central African Republic							164	53	54	68
Chad							630	22	24	53
Comoros							26	76	82	92
Congo							167	92	94	93
Cote d’Ivoire							838	57	59	91
DR of Congo							3,217	80	80	88
Equatorial Guinea							29	67	68	91
Eritrea							175	34	34	89
Ethiopia							3,176	16	16	41
Gabon							51	90	89	95
Ghana							884	73	71	91
Guinea							460	40	45	85
Guinea-Bissau							68	44	45	92
Kenya							1,571	61	62	96
Lesotho							61	77	78	95
Liberia							156	56	61	96
Madagascar							831	38	44	82
Malawi							665	89	87	96
Mali							758	45	49	70
Mozambique							1,087	55	54	91
Niger							983	59	40	83
Rwanda							363	91	91	99
Seychelles							2	-	-	-
Sierra Leone							229	54	60	97
South Africa							1,111	95	94	97
South Sudan							446	-	19	62
Swaziland							38	88	88	99
Togo							256	73	59	73
Uganda							1,665	57	57	93
United Republic of Tanzania							2,064	50	49	88
Zambia							645	67	64	96
Zimbabwe							539	80	80	94

1 Coverage estimates are derived from WHO UNICEF Joint Reporting (updated July 2016) http://apps.who.int/immunization_monitoring/globalsummary/timeseries/tswucoveragehepb_bd.html.

2 Annual birth data is derived from the WHO Immunization Monitoring System (updated May 2016) http://apps.who.int/immunization_monitoring/globalsummary.

3 Data derived from UNICEF (updated February 2016) www.data.unicef.org. SBA-skilled birth attendant, ANC-Antenatal care.

4 2015 coverage data reported to SAGE 2016 in WHO review of hepatitis B birth dose.

5 Sao Tome and Principe and Mauritius do not offer the birth dose universally, but follow a selective policy where infants of mothers that test HBsAg are offered vaccine.n.a.-not available.

**Figure 1:**
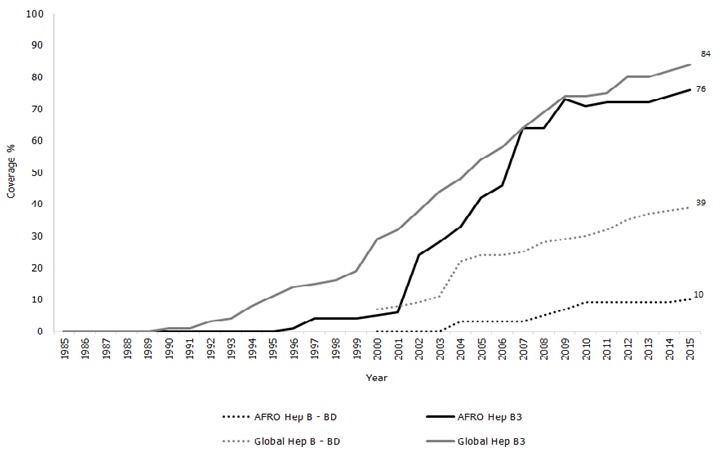
Childhood hepatitis B vaccination coverage in the World Health Organization African Region compared with global coverage, 1985-2015

Regional reported coverage with 3 doses of HepB (HepB3) increased from 5% in 2000 to 76% in 2015. However, coverage has plateaued at 70%-75% since 2009 (Figure 1) [11]. This is below the 2015 global HepB3 coverage of 84%. Country-specific HepB3 coverage estimates for 2015 ranged from 16% in Equatorial Guinea to 98% in Rwanda, The Seychelles, Swaziland, and United Republic of Tanzania; 16 (34%) countries reported national HepB3 coverage of at least 90% (Table 1) [4]. Regional reported HepB-BD coverage increased from 0% in 2000 to 10% in 2015, although coverage has plateaued at 10% since 2010 [12]. This is below the 2015 global HepB-BD coverage of 39% (Figure 1). Among countries that have introduced the birth dose, HepB-BD coverage ranged from 19% in Angola to 99% in Algeria and Botswana (Table 2) [5]. Algeria, Botswana, Cabo Verde, and The Gambia, all of which had introduced the birth dose over a decade ago, reported at least 90% national HepB-BD coverage (Table 2).

A recent situational report of the WHO African Region indicated HepB-BD introduction has been recommended or is under consideration in Cameroon, Cote d’Ivoire, Guinea Bissau, Mozambique, Niger, the Republic of Congo, Sierra Leone, South Africa, and Uganda [10]. In Ethiopia and Gabon, HepB-BD introduction has been proposed for the next comprehensive multi-year plan. In Rwanda, the national Expanded Programme on Immunization (EPI) reported that it has received approval from the Ministry of Health but is waiting for endorsement from the Interagency Coordination Committee (ICC). Ghana has included HepB-BD introduction in its comprehensive multi-year strategic plan for immunization and the National Viral Hepatitis Control Plan, but so far, HepB-BD introduction has been postponed due to competing priorities [10]. Countries have reported multiple barriers to HepB-BD introduction, including lack of financial support from Gavi, the vaccine alliance (10 countries), the need for evidence on the burden of chronic HBV infection and the risk of perinatal transmission in Africa (6 countries), insufficient cold chain storage (3 countries), lack of trained healthcare workers (HCWs) to attend births or conduct post-natal visits (2 countries), and a high proportion of home births (2 countries) [10].

### Impact of hepatitis B vaccination in children

To document the impact of HBV vaccination on chronic HBV infection prevalence in the African Region, we identified HBsAg serosurveys conducted pre- and post-HepB introduction among children in seven countries: the Gambia, Nigeria, Cameroon, Ghana, Senegal, South Africa, and Tanzania (Table 3) [13-36]. All studies were limited to a few areas, districts, villages, hospitals, or clinics, resulting in HBsAg prevalence estimates that were not representative of the true burden of chronic HBV infection among children in those countries. Some studies that were conducted post-HepB introduction, only included children that had three documented doses of HepB [17, 18, 21, 28, 33]. These studies reported HBsAg prevalence among fully vaccinated children, but they do not reflect the true burden among these age groups.

**Table 3:** Hepatitis B surface antigen seroprevalence among children pre- and post-vaccine introduction by country --- World Health Organization African Region

	Pre-Hepatitis B vaccine introduction					Post- Hepatitis B vaccine introduction						
Country	Year of study	Sample size	Study population	Age groups	HBsAg prevalence%	Year of study	Sample size	Study population	Age groups	HBsAg prevalence%	% with HepB history^1^	HepB3 coverage
**Countries with universal birth dose at the time of the surveys**												
Gambia^2^	1988	313	2 sets of villages on the north bank of the river[13]	3-8yrs	8	1998-99	235	Manduar and Kenebavillages [17]	1-<5yrs	1.3	100	100
	1988	959	7 neighboring villages on the south bank of the river[14]	6mo-5yrs	14.6	2003	236	Manduar and Kenebavillages[18]	1-4yrs	0	100	100
	1990-91	816	4 areas, 1 from each ecological zone in the country [15]	3-4yrs	12.6	2007-08	1921	40 villages in the Central River region [19]	1-5yrs	0.2	n.a.	-
	1995-96	823	4 areas, 1 from each ecological zone in the country[16]	9yrs	12							
Nigeria^3^	1997	200	Daycares and vaccination centers in Calabar, Ibadan, Warri cities [20]^4^	1-3yrs	20	2001	223	Sabondigga-Ora town[21]	1-4yrs	1.3	100^5^	100
	2001	219	Sabondigga-Ora town [21]	1-4yrs	4.6	2006	449	Sabondigga-Ora town [22]	6-8yrs	2	100^6^	-
	2006	373	Sabondigga-Ora town[22]	6-8yrs	11.8	2011	142	1 hospital; Benin city [23]^5^	2mo-<10yrs	14.1	27^7^	-
						2011	192	1 hospital; Ibadan[24]	<10yrs	0.5	81^8^	-
**Countries without universal birth dose at the time of the surveys**												
Cameroon	1989	172 530	6 primary schools in Kumba city [25]	4-6yrs 7-14yrs	17.4 25.1	2009-10	763	2 hospitals in Yaounde[26]	3mo-6yrs	0.7	23^9^	-
Ghana	1994	140 503	14 communities in the rural district of Ashanti- Akim North [27]^10^	1-5yrs <11yrs	9.3 21.7	2009-10	104	KissenaNankana district [28]	1-10yrs	1.9	100	100
						2013	433	11 health districts in Offin River Valley [29]	≤11yrs	1.8	n.a.	-
Senegal	1993	211	Kolda[30]	12-24mo	10.9	1993	229	Kolda[30]	12-24mo	3.9	86	40
	1999	2009	Health centers, nurseries, pre-schools, orphanages in Dakar &Thies[31]	0-<5yrs	13.5	2009-10	485	2 hospitals in Dakar [26]	3mo-6yrs	0.2	_43_ ^11^	-
South Africa	1995-96	2288	7 rural and urban clinics in Mdantsane district [32]	0-6yrs	10.4	1999	756	rural clinics, all provinces [33]	18mo	0.13	100	100
						2011-12	209 154	1 hospital, Pretoria [34]	1-5yrs 6-10yrs	0.5 1.3	n.a.	-
Tanzania	1991-92	199	General hospital in 1 urban, 1 rural area, Dodom[35]	1-5yrs	2.1	2006-08	110	Vaccination clinics at 2 hospitals, Moshi [36]	0-<5yrs	0	n.a.	-

HBsAg-Hepatitis B surface antigen, %-percent, HepB-hepatitis B vaccine, HepB3-3 doses of HepB, yrs-years, mo-months, n.a.-not available.

1 Document verified vaccination history.

2 Hepatitis B vaccine was introduced to Gambia progressively during the Gambian Hepatitis Intervention Study between 1986 and 1990.

3 Vaccine was widely available in Nigeria from 2004, but had been available in limited areas beforehand.

4 Information on the time period in which the study was conducted was unavailable, year of publication is presented.

5 52% (n=116) of children had also received the hepatitis B vaccine birth dose (HepB-BD).

6 All participants received ≥2 doses of HepB; 42% (n=188) of children had received the HepB-BD and an additional 3 doses of HepB.

7 All of the participants with documented vaccination history had received the HepB-BD, most (>68%) after 7 days post-birth. The proportion that received additional doses of HepB was not reported.

8 All of the participants with documented vaccination history had received HepB, but the number of HepB doses received was not specified.

9 99% of participants with documented vaccination history had received HepB, but the number of doses received was not specified.

10 Included compounds were selected based on the presence of anHBsAg-positive child from a previous school base serosurvey, therefore HBsAg prevalence estimates are likely to be over-estimates.

11 100% of participants with documented vaccination history had received HepB, but the number of doses received was not specified.

#### Countries that have introduced the birth dose

In The Gambia, surveys conducted between 1990 and 2008, most of which were associated with the Gambia Hepatitis Intervention Study (GHIS) an initiative which progressively introduced HepB into the Gambian routine immunization program during 1986–1990, showed that HBsAg prevalence decreased from 8%-14.6% among 6 month–9 year old children to < 2% among 1–5 year old children post-HepB introduction (Table 3) [13-19]. However, two of the three post-HepB introduction studies were conducted among children that had received at least 3 doses of HepB verified by vaccination records [17, 18]. Therefore, the true HBsAg prevalence may be higher. Available data from convenience and non-representative samples in Nigeria also found a decrease in HBsAg prevalence among children after vaccine introduction (Table 3) [20-24]. However, in three of four post-HepB introduction studies that found HBsAg prevalence to be < 2%, the majority of participants had documented HepB receipt [21, 22, 24]. In the remaining study, hepatitis B vaccination history was only available for 27% of the participants, thus some of the participants without vaccination history may not have received HepB, which is likely given national HepB3 coverage was 46% in 2011 in Nigeria (Table 1), resulting in the higher HBsAg prevalence of 14.1% [23].

#### Countries that have NOT introduced the birth dose

Studies conducted in Cameroon, Ghana, Senegal, South Africa and Tanzania, countries that did not have a HepB-BD in their schedule at the time of the surveys, reported a drop in HBsAg prevalence among children born after HepB introduction (Table 3) [25-36]. However, none of the studies were nationally representative. More specifically, estimated HBsAg prevalence among children born post-HepB introduction was less than 2% in Cameroon, Ghana, South Africa, and Tanzania [25-29, 32-36]. These estimates were derived from studies that only included children with document verified 3 doses of HepB (Ghana, South Africa), where documented vaccination history was only available for a small proportion of participants (Cameroon), or vaccination history was not reported (Ghana, South Africa, Tanzania). In Senegal, HBsAg prevalence was > 2% in a 1993 post-HepB introduction study [30]. This study was a cluster survey conducted a year after HepB introduction in a pilot region; hepatitis B vaccination history was available for 86% of participants, of which only 40% had received 3 doses of HepB. In comparison, another study conducted in 2009 reported a HBsAg prevalence of 0.2% among hospitalized children at one hospital in Dakar; hepatitis B vaccination history was available for 43% of participants and all had received HepB [26]. Since at the time of the latter study, national HepB3 coverage would have been around 90% (Table 1) it is likely that most of the participants without vaccination history were vaccinated.

### Evidence for perinatal transmission of HBV infection in Africa

### HBsAg and HBeAg prevalence among pregnant women

Understanding the prevalence of HBsAg and HBeAg among pregnant women or women of child-bearing age helps to assess the risk of perinatal HBV transmission and the need for a HepB-BD. From 1995 to 2016, we identified 75 studies from 18 countries that reported HBsAg prevalence among pregnant women (Table 4) [37-111]. Of these, 24 reported HBeAg prevalence [37-39, 45-47, 61, 70-72, 85, 88-90, 96, 100-102, 104-106, 108, 109, 111]. Over one third of the studies were conducted in Nigeria. Nearly all studies were cross-sectional or cohort designs that recruited participants through convenience sampling, many times at a single study site, and were subject to selection bias. Reported median HBsAg prevalence by country varied widely, from 1.9% in Madagascar to 16.2% in Niger (Table 4). Where reported, median HBeAg prevalence among HBsAg positive pregnant women ranged from 3.3% in Zimbabwe to 28.5% in Nigeria (Table 4). For most of the countries with multiple studies, HBsAg and HBeAg prevalence estimates varied widely, reflecting the different HBV infection risks in different parts of each country and among distinct population groups, e.g. groups of differing ethnicities, socio-economic status and education levels. Although none of the studies were nationally representative, they highlight that the prevalence of chronic HBV infection among the pregnant women surveyed was intermediate to high, and that the proportion of chronically-infected mothers who were HBeAg-positive varied widely by country.

**Table 4:** Hepatitis B surface antigen and e antigen prevalence among pregnant women by country within the World Health Organization African Region, 1995-2016

Country	No. of studies	Year of studies	Study site (no. of studies)		Study settingi ^1^(no. of studies)		Median No. participants per study (min, max)	Median % HBsAg prevalence (min %, max %)	Median % HBeAg prevalence among HBsAg positive women (min %, max%)
	(n=75)		Single	Multiple	Urban	Rural			
Western Africa	(n=46)								
Benin	1	2011	1	-	-	1	283	15.5 [37]	11.4 [37]
Burkina Faso	7	1995-2009	4	3	7	1	321 (129, 917)	8.1 (5.8, 17.1) [38-44]	21.2 (18.2, 24.1) [38, 39]
Cote D'Ivoire	3	1995-2002	1	2	3	-	498 (395, 4385)	8.0 (8.0, 18.2) [45-47]	14.5 (7.5, 15.3) [45-47]
Ghana	4	2000-2013	3	1	2	2	772 (168, 1368)	12.0 (9.5, 14.5) [48-51]	
Mali	2	1994-2009	1	1	2	-	2244 (829, 3659)	11.8 (8.0, 15.6)(52, 53]	-
Mauritania	1	2008-2009	-	1	1	-	1020	10.7 [54]	-
Niger	1	2008	-	1	1	1	495	16.2 [55]	-
Nigeria	26	1997-2015	20:	6	23	3	358 (150, 5760)	6.9 (1.4, 16.5)[56-81]	28.5 (6.5, 36.4) [61, 70-72]
Sierra Leone	1	2005	-	1	1	-	302	6.3[82]	
Central Africa	(n=8)								
Cameroon	7	2000-2015	1	6	4	5	349 (176, 7069)	7.7 (4.4, 20.4) [83-89]	12.1(0, 22.7) [85, 88, 89]
Gabon	1	2005	-	1	1	-	1186	9.2 [90]	10.1 [90]
Eastem and Southern Africa	(n=21)								
Ethiopia	9	2002-2015	6	3	8	1	269 (165,493)	4.4 (3.0, 7.8) [91-99]	12.5 [96]
Kenya	1	2001-2002	-	1	1	1	2241	9.3 [100]	8.8 [100]
Madagascar	1	2012	1	-	1	-	1050	1.9 [101]	5 [101]
South Africa	4	1999-2013	1	3	4	3	1124 (294, 1882)	3.9 (0.4, 5.8) [102-105]	17.1(0, 37.5) [102, 104, 105]
Uganda	1	2012-2013	-	1	1	-	397	11.8[106]	14.9[106]
Tanzania	3	1995-2010	2	1	3	1	434 (310, 980)	4.2 (3.9, 6.3) [107-109]	12 (O, 24)(108, 109]
Zimbabwe	2	1996-2005	1	1	2	1	701 (418, 984)	14.2 (3.3, 25.0)[110, 111]	3.3 [111]

No.–number; min–minimum; max–maximum; %-percent; HBsAg–Hepatitis B surface antigen; HBeAg–Hepatitis B e antigen.

1 If multi-site studies included both urban and rural settings they were recorded in both columns.

### Perinatal transmission of HBV in the African region

In addition to serosurveys conducted among pregnant women, we identified four studies that assessed perinatal transmission of HBV infection [38,45,49,108]. Among women of unknown or negative HIV status in Burkina Faso, Cote d’Ivoire, and Ghana, perinatal transmission of HBV was more frequent when mothers expressed HBeAg or had a high HBV DNA viral load (≥10^4^ IU/ml) [38, 45, 49]. In Cote d’Ivoire, 9 (38%) of 24 infants born to HBsAg-positive/HBeAg-positive mothers tested HBsAg-positive at six weeks of age, compared with none (0%) of 142 infants born to HBsAg-positive/HBeAg-negative mothers [45]. In Ghana, 5 (5.2%) of 97 infants born to HBsAg-positive mothers tested HBV DNA positive at two weeks of age; the relative risk of perinatal transmission associated with high maternal HBV DNA viral load (≥10^4^ IU/ml) compared with low maternal HBV DNA viral load was 2.4 (95% CI:1.1–5.4, p=0.048) [49]. One study from Burkina Faso reported that 7 (32%) of 22 infants born to HBsAg-positive/HBeAg-negative and 2 (29%) of 7 infants born to HBsAg-positive/HBeAg-positive mothers tested HBsAg-positive within 24 hours of birth [38].

### Challenges and strategies for improving hepatitis B vaccine birth dose coverage in Africa

Despite the introduction of HepB by all countries in the Region, for 31 countries (66%) HepB3 coverage is below the 90% recommended coverage level. Given the high chronic HBV infection prevalence throughout the Region, particularly among pregnant women, and the importance of perinatal and early childhood transmission in intermediate and high endemicity settings, countries need to improve HepB3 coverage and those without a birth dose might need to consider introducing the HepB-BD to reach the regional hepatitis B control goal by 2020. In African countries that have already introduced the HepB-BD, several challenges, including timely administration of the HepB-BD, high prevalence of home births, the lack of services available to reach infants born at home and unreliable vaccine supply have limited HepB-BD implementation. In this section we present those challenges and list some strategies that could help overcome them to improve HepB-BD coverage.

### Challenges associated with birth dose implementation in Africa

HepB-BD assessments conducted in the Region have consistently identified timely HepB-BD administration (within 24 hours of birth) as a challenge (BD workshop report, Regional Consultation on Viral Hepatitis Control, Mauritania CDC trip report, Botswana & Namibia BD assessments, unpublished reports). National policy recommendations for HepB-BD administration varied from within 24-hours in Nigeria to up to two weeks after birth in Namibia (Nigeria & Namibia BD assessments, unpublished reports). Restricting HepB-BD administration time to within 24 hours after birth, as recommended in Nigeria in February 2015, might limit coverage by preventing vaccination of infants born outside a health facility. In Nigeria, median timely HepB-BD administration was 1% (range: 0%–20%) and total HepB-BD coverage was 4% (range: 0%–22%) among health facilities visited prior to the birth dose assessment conducted in September 2015 (Nigeria BD assessment, unpublished report). In an assessment among children admitted to an emergency room in Nigeria, the mean age at HepB-BD receipt was 28 days, and only 13 (32%) of 41 infants received a HepB-BD within 7 days of birth [23]. In the Gambia, a review of 10 years of district HepB-BD vaccination coverage data showed that only 1% of infants were vaccinated within 1 day of birth, 5% were vaccinated within 7 days, and 58% were vaccinated within 28 days [112]. In Botswana, 50% of the facilities visited during a birth dose assessment administered the vaccine within 24 hours of birth (Botswana BD Assessment, unpublished report).

Other challenges identified included high prevalence of home births and the lack of services available to reach infants born at home, unreliable vaccine supply and inappropriate forms to document HepB vaccination (BD workshop report, Regional Consultation on Viral Hepatitis Control, Mauritania CDC trip report, Botswana & Namibia BD assessments, unpublished reports). In The Gambia and Nigeria, where the proportion of home births was high, cultural factors such as waiting until after a child’s naming day (around 7 days) to bring him/her to a healthcare facility delayed vaccination (BD workshop report, Botswana & Nigeria BD assessments, unpublished reports). Vaccine stock outs or limited vaccination sessions hindered the provision of timely HepB-BD in Botswana, The Gambia, Mauritania, Namibia, and Nigeria (BD workshop report; Botswana, Namibia, & Nigeria BD assessment, Mauritania CDC trip report, unpublished reports) [112]. In Botswana, despite daily HepB immunization sessions, HepB stock outs lasting over one month were reported at two of 16 visited facilities; and in Namibia, two events of HepB stock outs were reported in the six months before the assessment visit. Monitoring HepB-BD coverage is dependent upon having appropriate documentation tools to record both timely and total HepB-BD coverage. For all assessed countries, documentation of HepB-BD administration was suboptimal (BD workshop report; Botswana, Namibia, Nigeria, Sao Tome and Principe BD assessments; unpublished reports). In Nigeria, only doses administered within 24 hours could be recorded, while in most other countries there was no place to record timely HepB-BD.

### Strategies for improving birth dose coverage

Many of the challenges identified in the HepB-BD assessments in Africa can be overcome based on the experiences in the WHO Western Pacific Region (WPR) and South East Asian Region (SEAR), where several countries had high HBsAg endemicity and high home birth rates. By implementing hepatitis B vaccination strategies, including HepB-BD administration, the WPR decreased chronic HBV infection prevalence among children at least 5 years of age from 8.3% in 1990 to 0.9% in 2012 [113]. The strategies described below could help to improve HepB-BD coverage and promote the achievement of the hepatitis B control goal in the Africa Region.

#### Advocate for strong political commitment

Strong political commitment is essential to identify resources in the country’s budget or to seek financial support from donors to introduce HepB-BD. In order to engage decision makers, initial steps need to be taken, including gathering the evidence on the prevalence of chronic HBV infection in pregnant women and risk of perinatal transmission, to present to the national immunization technical advisory group or equivalent technical bodies within each country to review [114]. All potential decision makers and opinion leaders from a wide variety of organizations should be engaged, including Ministries of Health and Finance, professional societies, medical associations, donor agencies, non-governmental organizations, as well as community and religious leaders [114]. Advocacy with partners in different but related sectors, such as cancer prevention, chronic disease prevention, safe motherhood and essential newborn care, might also strengthen political commitment [114]. It will be important to highlight that monovalent HepB is affordable, varying from US $0.16 per dose for a 10-dose vial to US $0.38 per dose for a single dose vial [115]. In addition, the vaccine is 95% effective in preventing infection in newborns [1].

#### Develop clear policy recommendations

HepB-BD guidelines and policies should be consistent with the WHO Strategic Advisory Group of Experts (SAGE) recommendations. All infants should receive their first dose of HepB as soon as possible after birth, preferably within 24 hours and up until the time of the first primary dose, since vaccination up until 7 days after birth can still be effective at preventing perinatal HBV transmission [116]. Infants born to HBsAg-positive mothers vaccinated after 7 days post-birth, compared with those vaccinated 1-3 days after birth, had an increased risk of HBV infection (OR = 8.6) [1]. HepB-BD administration is needed for all infants, because selective vaccination of infants born to HBsAg-positive mothers identified by screening as is the current policy for Sao Tome and Principe and Mauritius, has been found to miss at-risk babies [117].

In China, an effective policy to ensure timely administration of the HepB-BD was to assign responsibility for vaccine administration to whoever delivered the infant (“Who delivers the infant gives the birth dose”) [118].

All countries providing or considering HepB-BD introduction should ensure that staff at health facilities, hospitals, and public health departments, including MCH and perinatal care staff, are well-trained on the policy recommendations and reporting requirements to help address challenges that have been identified during the HepB-BD assessments.

#### Ensure relevant documentation for monitoring birth dose administration is available

To appropriately monitor HepB-BD coverage, countries need to record separately birth dose administered within 24 hours of birth and birth dose provided after 24 hours. Countries in the Africa Region that have introduced the birth dose and were recently assessed, had insufficient tools for documenting both timely and total birth dose. Therefore, all immunization reporting tools, including immunization cards, EPI registers, and electronic data management systems, should have a place to record the date of HepB-BD administration and to track whether it was provided within 24 hours of birth. Synergy of data collection forms across EPI and Maternal and Child Health (MCH) programs could also improve data monitoring.

#### Maximize HepB-BD coverage among health facility births

In just under one third of countries (14 out of 47) in the Region, ≥ 80% of births occurred in health facilities (Table 2). In these countries, high HepB-BD coverage can be achieved among children born in health facilities. Utilizing ANC visits to promote health facility deliveries could improve post-natal care and maternal health as well as facilitate administration of HepB-BD. In 81% of countries (38 out of 47) in the WHO African Region, ≥ 80% of pregnant women had at least one ANC visit and in 57% of countries (27 out of 47), ≥ 90% of pregnant women had at least one ANC visit (Table 2). Also, engaging local community and religious leaders to promote hospital births may be helpful. In China, increasing hospital deliveries resulted in improving timely HepB-BD coverage and contributed to decreasing maternal mortality, and eliminating maternal and neonatal tetanus [118].

Appropriate training for MCH and EPI staff is also essential and can improve timely HepB-BD coverage. In the Philippines, assessment and correction of HCWs through on-the-spot training increased timely HepB-BD coverage among hospital births [119]. In China, strong collaboration between MCH and EPI programs, supervision of low performing sites, and HCW training led to high timely HepB-BD coverage among hospitals [118]. Further improvements could be made through well managed vaccine delivery and procurement to avoid vaccine stock outs at health facilities. Availability of HepB-BD policies on-site and standing orders for birth dose vaccination led to higher HepB-BD coverage in the Philippines [119].

#### Reach children born at home within 24 hours of birth

Across Africa, one of the major challenges for timely administration of the HepB-BD is the large proportion of home births. In nine countries, institutional births accounted for ≤ 50% of births (Table 2). In settings where births are attended by an SBA, the HepB-BD can be administered by SBAs who have been trained to administer monovalent HepB and provided access to the vaccine. In the African Region, SBAs attend > 70% of births in 18 countries (Table 2).

In settings where births are not attended by SBAs, activities that could improve timely HepB-BD coverage include tracking pregnancies to increase HCW awareness of potential births in their community, educating pregnant women about the importance of timely HepB-BD administration during antenatal care visits and training community health workers (CHWs) or volunteers to organize outreach visits to vaccinate the newborn in a timely manner. Birth notification using village health volunteers was piloted in Lao PDR and resulted in more health facility deliveries and improved HepB-BD coverage [120]. Training CHWs or traditional birth attendants (TBAs) to vaccinate newborns with compact single-dose pre-filled auto-disable injection devices (CPAD) of monovalent HepB (Uniject™) has been conducted in Indonesia to provide HepB-BD during outreach activities for home births, improving timely access to the vaccine [121].

#### Storage of HepB outside the cold chain

A strategy for reaching newborn infants in areas that lack or have unreliable cold chain is to store monovalent Hep B outside the cold chain (OCC). Data from several HepB manufacturers indicate that the vaccine is thermostable for at least 4 weeks at temperatures of 37°C and 40°C–45°C and that HepB stored OCC induces a similar level of protection and seroconversion as vaccine stored between 2°C–8°C. Recently, WHO SAGE issued recommendations that support countries that choose to pursue an OCC policy for monovalent HepB vaccination [116, 122]. Storing monovalent HepB OCC has been shown to significantly improve HepB-BD coverage in Indonesia, China, Lao, and Vietnam [123-126]. Therefore, countries in the African Region, where the proportion of home births is high and/or cold chain storage might not be optimal, might want to consider storing monovalent HepB OCC to promote timely HepB-BD administration and subsequently prevent perinatal infections.

## Conclusion

Substantial progress has been made to introduce routine infant hepatitis B vaccination in the WHO African Region; however, in 2015 the Region had the lowest HepB3 coverage estimate (76%) compared with other WHO Regions [11]. Only 11 (23%) countries have introduced the Hep B-BD; of these, five had total HepB-BD coverage ≥ 80%.Limited data from non-representative serosurveys conducted to date suggest hepatitis B vaccination has decreased HBsAg prevalence among children born after HepB introduction in some countries. However, several studies reported > 2% HBsAg prevalence among children born after vaccination introduction. Given the HBsAg prevalence data among pregnant women and children is limited and not nationally representative, the prevalence estimates presented for each country may not reflect the true situation. High quality nationally representative serosurveys among children born after implementation of routine infant hepatitis B vaccination are needed to inform local decision makers about progress towards and actions needed to achieve the Africa Region hepatitis B control goal. National estimates of HBsAg prevalence among children could be obtained by conducting nationally representative hepatitis B serosurveys or by incorporating HBsAg testing into Demographic and Health Surveys, Multiple Indicator Cluster Surveys, or HIV/AIDS surveys. Available data suggest that a substantial burden of chronic HBV infection among children might be occurring from perinatal and early childhood HBV transmission in the African Region. These findings reinforce the need to improve HepB3 coverage and that some countries might need to consider introducing a HepB-BD to achieve the regional hepatitis B control goal. Ultimately, all countries will need the HepB-BD to make further progress to eliminate mother to child HBV transmission by 2030 [127]. To facilitate HepB-BD introduction and improve timely HepB-BD coverage, strategies are needed to reach both facility-based and home births. Strong political commitment, clear policy recommendations, and training of staff on HepB-BD administration and recording are also required

### What is known about this topic

- There is a high prevalence of chronic hepatitis B virus (HBV) infection in the African Region, resulting in 100 million people being at risk of death from liver cancer and cirrhosis;
- The Africa Region has established a hepatitis B control goal to reduce the prevalence of hepatitis B surface antigen (HBsAg, a marker of chronic HBV infection) to < 2% among children aged < 5 years by 2020;
- Administering a hepatitis B vaccine birth dose within 24 hours of birth and three additional doses during infancy protects children from acquiring chronic HBV infection through perinatal transmission and during early childhood when the risk of developing chronic infection is highest.

### What this study adds

- All countries in the Africa Region have introduced hepatitis B vaccine in the routine childhood vaccination schedule, but only 11 have introduced the birth dose;
- Childhood hepatitis B vaccination has reduced chronic disease among children; however, several studies documented > 2% HBsAg prevalence among children born after vaccine introduction, highlighting the need for countries to improve HepB3 coverage and to consider introducing HepB-BD to prevent perinatal and early childhood HBV transmission;
- High quality nationally representative serosurveys among children born after the implementation of routine infant hepatitis B vaccination are needed to inform decision makers about progress towards and actions needed to achieve the African Region hepatitis B control goal.

## Competing interests

The authors declare no competing interest.
